# Towards health equity: core components of an extended home visiting intervention in disadvantaged areas of Sweden

**DOI:** 10.1186/s12889-022-13492-3

**Published:** 2022-06-01

**Authors:** Madelene Barboza, Anneli Marttila, Bo Burström, Asli Kulane

**Affiliations:** 1 Department of Global Public Health, Equity and Health Policy Research Group, Karolinska Institutet, 17177 Stockholm, Sweden; 2 Department of Public Health and Sports Science, Faculty of Occupational and Health Sciences, University of Gävle, Kungsbacksvägen 47, 80176 Gävle, Sweden; 3 Region Stockholm, Centre for Epidemiology and Community Medicine, Box 45436, 104 31 Stockholm, Sweden

**Keywords:** Early childhood development, Home visiting, Health equity, Proportionate Universalism, Core components, Qualitative analysis

## Abstract

**Background:**

Understanding the mechanisms of implementation of public health interventions in community settings is a key aspect of programme assessments. To determine core components and establish a programme theory are important tools to improve functioning and support dissemination of programme models to new locations. An extended early childhood home visiting intervention has been developed on-site in a socioeconomically disadvantaged area of Sweden since 2013 with the aim of reducing persisting health inequities in the population. This study aimed at investigating the core programme components and how the intervention was perceived to contribute towards health equity from early childhood.

**Methods:**

Qualitative framework method was applied in a document analysis and subsequent semi-structured interviews with 15 key actors involved in the programme.

**Results:**

The intervention was found to be constituted of five core components centred around the situation-based, parental strengthening work method delivered by a qualified team of child health care nurse and social worker. The programme theory foresaw positive effects on child and parental health, responsive parenting practices, families’ use of welfare services according to need and increased integration and participation in society. The principles of Proportionate Universalism were recognised in the programme theory and the intervention was perceived as an important contribution to creating conditions for improved health equity for the families. Still, barriers to health equity were identified on the structural level which limit the potential impact of the programme.

**Conclusions:**

The core components of the Extended home visiting programme in Rinkeby correspond well to those of similar evidence-based home visiting interventions. Combining focus on early childhood development and responsive parenting with promoting access to the universal welfare services and integration into society are considered important steppingstones towards health equity. However, a favourable macro-political environment is required in the endeavour to balance the structural determinants’ influence on health inequities. Improved availability and accessibility to welfare services that respond to the needs of the families regarding housing, education and employment are priorities.

**Trial registration:**

The study was retrospectively registered on 11/08/2016 in the ISRCTN registry (ISRCTN11832097).

**Supplementary Information:**

The online version contains supplementary material available at 10.1186/s12889-022-13492-3.

## Background

### Interventions for healthy early childhood development

Advances in research in the past decades have firmly established the importance of healthy early childhood development (ECD) in a life course perspective [1–4]. It is also well understood that health and development for all children is priority in combatting health inequities within societies [5–7]. Despite this knowledge, health inequities persist, also in welfare states such as Sweden. With the pronounced aim of contributing to equal and just child health care (CHC) for all children in Sweden, the Swedish universal CHC programme reaches virtually all children between 0 and 6 years of age [8]. It is composed of one home visit by a CHC nurse when the child is new-born, one more home visit at eight months, implemented in some regions, and approximately 15 visits at the CHC centre, four of which count on the participation of a paediatrician in addition to the nurse. There is a universal health promotive and preventive content which is offered to all, as well as specific actions and resources that may be offered to any child and family at any point in time when extra needs emerge. Even with the comprehensive national CHC programme, systematic differences in health are observable from an early age, as can be seen in the annual report of CHC Stockholm region, covering 25% of all Swedish children, where significant geographical differences are shown in the indicators of child health, closely tied to the socioeconomic status of the population [9].

Along with the growing understanding of the importance of ECD, interventions with this aim have been developed, tested and evaluated across different contexts. The scientific evidence of ECD interventions have been compiled into the Nurturing Care framework [10], declaring that the five components of good health, adequate nutrition, responsive caregiving, opportunities for early learning, and safety and security, are essential to the child. The involvement of caretakers, community and service networks are of great importance and interventions should be multisectoral and comprehensive, preferably made up of a combination of services targeting child and parents [3, 4, 10]. Extended home visiting during early childhood is one intervention which is recommended [3, 4] and has increasingly been adopted in high income countries. Reviews have indicated both positive [11–17] and more modest [18–20] effects and recent developments of research and initiatives in the home visiting field are thus focusing on how to strengthen programme capacities. One example is the field of precision home visiting that attempts to discern what programme components work for whom, where and how, to tailor interventions and thus improve effects as well as family retention [21–24].

### The Rinkeby extended home visiting programme

In 2013 a new home visiting intervention was developed in the district of Rinkeby, in Stockholm municipality. The socioeconomic and health indicators of its population placed the area as one of the most disadvantaged in the region [25, 26]. The high percentage of inhabitants with foreign background [27] was a further factor known to influence health inequities [28]. The nurses at the CHC centre experienced lack of time and resources to respond to the needs of the families with small children, while the social services in the area found it difficult to reach out with their preventive parenting support activities to families [29]. The new intervention was therefore based on a practical collaboration between CHC nurses and social workers (parental advisors) from the preventive branch of the social services. The team of professionals delivered six home visits during the first 15 months of life (the first general visit of the CHC programme + five extra visits), embedded in the process of the national CHC programme of centre-based services. This was an innovation in the Swedish context [30]. All first-time parents in the area were offered the intervention and over 90% participated [31]. The programme model was developed on site, in collaboration with a research team from Karolinska Institutet and with financial support from the Swedish Public Health Agency. The proposal to offer this extra support to all first-time parents in a disadvantaged area was based on the principles of Proportionate Universalism [32], with the aim to positively contribute towards the levelling of the social gradient in health [30]. The programme was integrated into the permanent activities in Rinkeby in 2017 and participation rates have remained above 90% [33]. The model has also been disseminated to 15 additional areas in Stockholm region as well as to several other regions in the country.

The intervention was developed with a high degree of involvement of the CHC nurses and parental advisors. The professionals decided not to engage in any initial training but to gradually develop content and work methods as the intervention was rolled out. The content of the national CHC programme served as an overarching framework and the parents own questions, interests and worries directed the delivered content of each individual visit [29]. The programme has been accompanied and evaluated since 2013 by Karolinska Institutet and the study protocol presents an initial basic programme theory [30]. In 2018 the CHC nurses and parental advisors themselves described the work method and main ECD themes of the visits in a programme guide, in Swedish, which is being used by new professionals entering the programme and also by the new programme sites that are adopting the model [29].

### Developing programme theories and discerning core components

Central in the efforts to improve interventions in early childhood is to gain detailed understanding of what they contain, how they are delivered and by whom, areas where there are still knowledge gaps in the ECD literature [10, 34]. In order to understand an intervention’s capacity to achieve its stated aims it is essential to develop a programme theory that spells out expected programme effects and the reasoning of how the intervention is supposed to generate these effects for the specific target audience [35, 36]. Involved in this effort is also to identify the core components, or active ingredients, of the intervention that are essential for producing the expected effects [21, 34]. Core components may include programme contents, delivery aspects or characteristics of the home visiting professionals [34]. In addition, it is also important to establish core components and mechanisms of the implementation process of an intervention to ensure improvements and successful scaling-up of ECD and home visiting programmes, an area where research is also still needed [35, 37–39]. Consistency between the programme theory, and the realisation of the intervention and implementation processes in practice may be understood as a central condition for the success of home visiting programmes [36].

Further, The World Health Organization’s Commission on the Social Determinants of Health, as well as the Swedish Commission on Health Equity, have stated the need for strengthening of the knowledge base on health inequities and potentially effective measures targeting the social determinants of health [5, 28]. The Swedish Commission specifically called for monitoring and evaluation of extended home visiting programmes in order to improve their development [28].

At this point in time, when the Rinkeby extended home visiting programme has been integrated into usual practice for four years and is also being implemented in several other areas, it appears important to investigate what constitutes core components of intervention and implementation and how the intervention is expected to contribute towards health equity, thus revising the original programme theory. The aim of this study was to contribute towards increased understanding of the core components of a multisectoral early childhood home visiting intervention carried out in socioeconomically disadvantaged areas of Sweden and explore how such an intervention may contribute to decreased health inequities in these areas.

## Methods

The study was developed with a qualitative approach in a two-phase process, composed of an initial document analysis followed by semi-structured interviews with key informants. The Framework method proposed by Gale et al. [40] was used when conducting the analyses. This method was chosen for its capacity to manage and give an overview of large amounts of data. It is also considered a flexible method which is not tied to any specific epistemological approach and can be used with deductive, inductive and combined types of analysis [40], something which suited the structure of the study.

The first phase of document analysis was understood to be an effective way of using already existing data to create a good initial understanding of the development of the intervention, its different components, the context and target group, as well as the implementation process. An analytical matrix was developed prior to the start of data analysis. The matrix was intended to contain a broad spectrum of intervention and implementation components into which the contents and characteristics of the Rinkeby extended home visiting programme could be mapped. For implementation components the Consolidated Framework for Implementation Research (CFIR) was used [41]. It is made up of five domains: Intervention characteristics; Outer setting; Inner setting; Characteristics of individuals; and Process, which contain 39 constructs and sub-constructs. In addition, it was considered important to include components specifically from the ECD and home visiting literature into the mapping exercise. Nine reviews and key articles were used (Additional file 1) and a list of 53 components in ten categories was developed and added to the matrix. All constructs and components in the matrix can be found in Additional file 2.

A total of 37 sources containing information on Rinkeby’s extended home visiting programme were identified, such as scientific research articles, evaluation reports, material produced by the professionals in the programme, reports from government commissions and public authorities, and media reporting, published between 2015 and 2021. The material contained text as well as recorded interviews in podcasts and video. For a complete list see Additional file 3. All material was read through and listened to for initial familiarisation. Each text source was then analysed line by line and the components from the matrix were indexed onto the relevant passages of text. The identified parts were charted into the matrix, either in its verbatim form or summarised, depending on the length of the excerpt. The recorded sources were listened to, and relevant passages were identified, transcribed, indexed and consequently chartered into the matrix, verbatim or in summarised form. Analysis was then carried out on the chartered data regarding each construct and component in the matrix and a written synthesis of the analysis was produced. This synthesis contained a summary of the information obtained for each construct and component, analysis of the meaning and relevance of the information as well as indications of information gaps and further questions that had arisen. The application of the Framework method was carried out by the first author and the results in terms of the matrix with chartered data and the written synthesis was discussed among all authors.

Based on the results from the first phase of the study a preliminary version of the programme theory was developed, containing information on target group, intervention activities and resources, expected short- and long-term effects as well as the overarching goals. An interview guide was also produced (available in Additional file 5). It contained questions that led the interview subjects to reflect on the composition and relevance of the proposed programme theory compared to their practical experience of the intervention, as well as the programme’s capacity to produce the effects and reach the goals stated in the theory. There were also questions related to what implementation components and processes were considered essential for the functioning of the intervention. Two pilot interviews were carried out which resulted in reformulation of some questions and shortening of the interview guide.

Through purposive sampling, 15 key informants with relevant knowledge of the home visiting intervention were identified for the interviews. All key informants accepted to participate. They were coordinators, local managers and higher managers from the CHC and social services, representing a broad experience from the different phases of development and implementation of the home visiting programme (see Table 1). Final sample size was determined by applying the concept of information power which assesses study aim, theoretical perspective, analysis method, access to relevant informants and information quality [42]. Individual interviews were carried out by the first author with 13 of the key informants and one joint interview was carried out with two key informants in accordance with their wishes. All interviews were carried out via a video-conference app with visual and sound recording. Verbal consent for participation was given by all participant and recorded prior to the interview. The preliminary programme theory was sent out before the interviews and was also shared on screen during the interview as a base for discussion. The interviews lasted between 39 and 85 min and were transcribed by the first author. All transcripts were read through twice for familiarization while analytical notes were written simultaneously.

**Table 1:** Profiles of 15 interviewed key informants

Area of experience	Nr of informants
Gradual development and start-up of the programme model in Rinkeby	6
Implementing the intervention in the geographical location of Rinkeby	9
Implementing the intervention in other geographical locations	9
Overview of general scale-up process of the intervention to all areas in Stockholm or another region	6

A tentative matrix was developed, and three transcripts were indexed according to the categories. The matrix was reviewed and revised, and all transcripts were subsequently indexed. The indexed data was then chartered into the matrix in summarised form. Each category in the matrix was analysed separately and a final analysis was written considering the complete matrix. In order to ensure dependability of the analysis, comparisons were made between the data from key actors who had worked during the early stages and those from more recent times, between key actors from different areas and professional groups but no significant variations were encountered. The indexing and chartering were carried out by the first author while the development of the matrix and the final analysis was reviewed and discussed within the group of authors after which some adjustments were made. The findings are presented in a final programme theory and an interpretative text containing three main themes. The analysis also produced detailed descriptions of the target audience and implementation components and conditions that are presented in Additional file 4.

The analysis process was continuously registered by the first author in analytic notes, which provided an audit trail of the process as well as the content on reflexivity. This material served as input for all discussions in the group of authors.

## Results

**Fig. 1:**
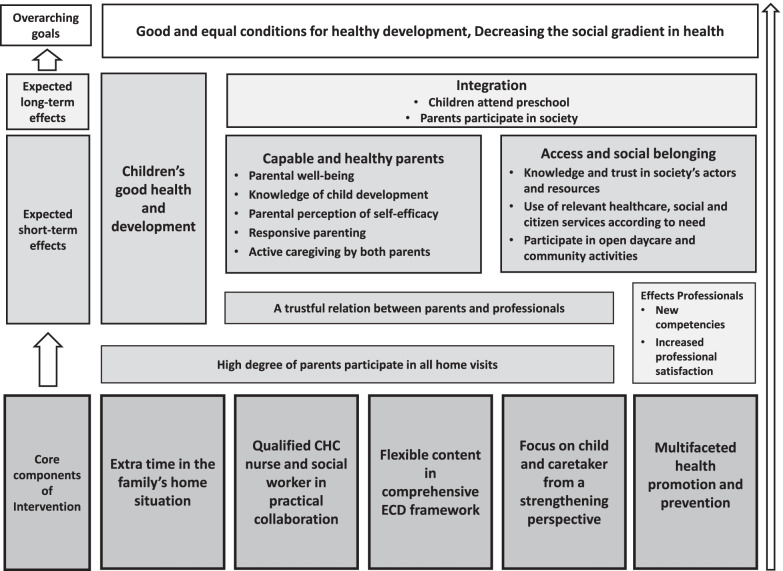
Programme theory of Rinkeby extended home visiting programme

The final programme theory in Fig. 1, contains a set of five core components of the intervention, expected short- and long-term effects as well as the overarching goals towards which the intervention should contribute. All parts of the programme theory are further developed through the three main themes of the analysis: Core components for a comprehensive and customized intervention; Expected effects of healthy children and parents integrated in the universal system; and A step on the way towards the goals of health equity.

### Core components for a comprehensive and customized intervention

#### Extra time in the family’s home situation

A key intention of the intervention is to offer the family more time with professionals on their own conditions. Making visits to the family's home represents a more equal meeting between parents and professionals, where the family feels safe and more relaxed. It gives possibilities for the professionals to observe the interplay between child and parents in their everyday environment and creates a better understanding of the family's situation and needs. The six visits of one hour over 15 months are interwoven with the standard 30 min-visits to the CHC centre within the CHC programme. For the nurse, the home visits are an opportunity to have more time to see to the family's needs within the frame of the CHC programme.

“With the home visits there is more space to build trust and to return to issues several times. You can spend more time on the subject and there is also more opportunity as a CHC nurse to see the family as a whole and not only, ‘this child is exposed to tobacco smoke’, but you also see, ‘yes, and the family has a trauma from escaping from this war and they have no housing right now and they are looking for a place in childcare’. So you see the whole picture in a totally different way.” (Key actor 5)

For the preventive social services, the six visits represent a unique opportunity to establish relations with families early.

“The CHC nurses have been carrying all worry about the families themselves, and the home visits is a way to add more eyes that see, and someone to talk to about the family. And that some of the challenges of the families end up where they belong, with the parental advisors and social services.” (Key actor 7)

#### Qualified team in practical collaboration

The intervention is carried out by a team of a specialist CHC or public health nurse and a social worker (parental advisors) from the preventive social services, both often with long professional experience. The two qualified professionals are perceived as key resources in delivering the visits and their practical collaboration in the family’s home is the central pillar of the intervention. The presence of two professions ensures the capacity to cover a broad range of content and respond to a diversity of needs. It also adds diversity to perspectives on ECD and parenting.

“There is an interplay between the CHC nurse and parental advisor, with their different competencies, that offers the parents a very wide spectrum of possibilities for reflection and advice, and that they are seen in many different ways.” (Key actor 2)

To collaborate in practice creates a multifaceted and deep understanding of each family and it qualifies the interactive dialogue with parents. The professionals also give support to each other in difficult and complex situations. The same team is maintained during the process to ensure stability for the family and to enable the building of a trustful relation that serves as a base for the intervention. It is additionally understood that a positive relation with the professionals will contribute towards trust in the CHC and preventive social services as institutions.

#### Flexible content in a comprehensive ECD frame

The principal thematic ECD content of the visits is drawn from the CHC programme and Nurturing Care framework. A balance is sought between the general content and the specific needs and wishes that are observed and presented during the meeting with each family. Thus, there is no set agenda for each meeting, but the professionals are required to observe the situation and adjust the content accordingly. This flexible work method which relies heavily on the competencies and experiences of the professionals is believed to be a strength of the intervention.

“Situation-based work makes it very apparent, that the situations can be so different, how you live, what the safety looks like, previous knowledge, what country you come from, there are so many factors, and that is what is so skilful in the approach, to be able to take in and consider all these situations in the short meeting, two professionals, and make the visit as meaningful as possible.” (Key actor 6)

#### Focus on the child and caretakers from a strengthening perspective

The child's development and well-being are at the centre of the intervention. The child's health, development process as well as interplay between child and parents are observed and discussed every visit. The professionals focus on the relation between the child and each of the parents as well as the relationship between the parents. Active participation by both parents (or caregivers) in the meeting is sought and continuous efforts are made to include fathers even when they are not physically present at the visit. The professionals acknowledge existing positive parenting strategies and aim to promote parenting self-efficacy. They seek to give support when needed while always encouraging parents’ own decision-making and initiatives.

“We work with strengthening what functions well, it is very important to have focus on this, ‘well done, carry on like that!’. Because it is a way to help parents with their worries of parenthood, it gives them energy. And maybe they also dare to bring up those things that don’t work, because they are not easy to talk about, because you feel the expectation of being a good parent. So that is an important part in this work.” (Key actor 14)

#### Multifaceted health promotion and prevention

The home visits embrace the promotion of health, well-being and development of the child as well as the health, psychosocial well-being and parenting self-efficacy of the parents. Health promotion enters the agenda through the Nurturing Care components of good health, nutrition, responsive caregiving and early learning. The professionals also guide parents to additional services in the community such as library, open daycare and language classes. Further, the professionals act with a preventive focus as well, for example by treating issues such as child safety and intrafamily violence. They identify and deal with families’ difficulties or extra needs.

“To reach them early and be able to help with those small, but so important things as early as possible. To care for their concerns before they become big problems, to prevent that they grow and become too heavy and difficult.” (Key actor 14)

The parental advisor can offer psychosocial support to parents during the visit or book a separate meeting for advice and support. Alternatively, information and guidance are given for parents to access relevant resources, such as healthcare, citizen services or social services.

The key actors confirmed a common understanding of the core components, and their delivery was not perceived to have been affected over time or by the variations of characteristics of the target group between the different programme sites.

### Expected effects of healthy children and parents integrated in the universal system

#### Accessing the core components to promote ECD

The first fundamental step towards effects in the programme theory is that all families accept and take part in the intervention.

“I think that this is the first part, that the family even accepts to participate and then they should receive all visits, and I think we have succeeded in describing how important this is. So this is really the base for everything, that we get them into the programme.” (Key actor 6)

A central expectation is then that the intervention components promote children’s well-being and healthy development. This is understood to be a direct result of the professionals’ actions during the home visits, but also indirectly through the following two areas of results that the intervention aims to positively affect.

#### Capable and healthy parents

The intervention should lead to parents having sufficient knowledge of children’s health and development and feeling confident in their parenting role. They are also expected to adopt responsive parenting practices such as interplay, language stimulus and play. The aim is to actively engage both parents in the responsive parenting practices. There is a clear understanding that parents’ good mental and physical health is essential to ensure parenting self-efficacy and practices, which in turn is vital for the health and development in the child. Parental health is also perceived to be an important condition for achieving the other foreseen results and thus the intervention aims to promote mental and physical health also with regards to the parents.

“We know that parents may need more knowledge on children’s development to perceive how competent the small child really is. And it is much easier to see that if you get knowledge and someone shows and helps you to see, and that will of course improve the interplay between parents and children. If you help parents perceive the signals from small children it will increase their attention towards the child, and their care becomes more responsive.” (Key actor 1)

#### Access, social belonging and integration

There are also expectations that information, guidance and a trustful relation with the professionals will result in parents’ knowing of available resources and overcoming thresholds such as language barriers and lack of trust, to access and use societal support services when they need.

“It feels like their trust in public authorities has increased. There are not that many who are sceptical. And I also perceive that there are more families who use preschool and other resources when they have participated in the home visiting programme, so they know what kind of help they can get.” (Key actor 9)

The assumption is also that the intervention contributes to the strengthening of a sense of social belonging, especially for isolated families, where parents and children start participating in open daycare and other community activities. Children entering regular preschool is seen as an important result following the intervention and parents’ integration and participation in society is an important goal towards which the programme expects to contribute.

“There is quite a high number of parents who actually came to the open daycare and participated, and we have a perception that this can lead to expanded social networks and that they take part in open community spaces in a different way. One of the CHC centres experienced very clearly an increase in their general parenting groups and many were parents from the home visiting programme.” (Key actor 12)

Additional results with regards to the professionals have been observed. The intervention provides opportunities for new skills development for both groups of professionals. Improved professional satisfaction has led to less turnover and greater facility in recruiting staff to the programme sites.

### A step on the way towards the goals of health equity

#### Creating good conditions

The overarching goals are to achieve good and equal conditions for healthy development, and to decrease the health gradient and it is perceived that the intervention provides a positive force in the right direction through several of the programme strategies. Strengthening parents is one central part.

“If you support parents who feel very lonely and not seen by anyone, when you give them confidence and let them know that they are good parents. And you give them information so they can take informed decisions about taking care of their children. And this type of information together with strengthened parenthood, I think will contribute towards decreasing inequalities.” (Key actor 5)

Another of the strategies is by working with a broad comprehensive scope.

“You can do this in different ways, but I believe that a perspective of health equality entails health promotion and prevention, that is what it is about. I believe we can decrease the difference and really promote health and prevent unhealth and prevent social vulnerability and exclusion.” (Key actor 1)

Finally, to adopt principles of universal support to a targeted group is acknowledged as important.

“Equal and good conditions for growth and development, that is when a whole group with less favourable prerequisites all receive this extra support. I believe we really achieve a lot with this model, especially in complement with other interventions. I believe this firmly.” (Key actor 2)

#### Health equity as a complex process

While the intervention is regarded as a possibility to create conditions for a better start to life, it is also recognized that this is only a first step in a longer process.

“Well, a child doesn’t turn 15 months and then stop. A child is a child until 18 years, so we can only lay the groundwork during the first 15 months. Then it is the parents who take over, and the next sector, like school, and authorities and so on.” (Key actor 6)

It is also understood that many challenges in the families’ life situations may influence health and well-being negatively. Structural determinants such as segregation, unemployment and crime remain large in disadvantaged areas and the intervention is understood to be only a limited force for change.

“I believe it [the intervention] can contribute towards a partial flattening of the social gradient in health, but it doesn’t influence society on the whole. It doesn’t influence the living conditions of families. If you want a society with equal and good conditions for growth and development, then you need to work with broader efforts than an extended home visiting programme.” (Key actor 13)

#### Availability and accessibility of services

Structural forces are also recognized to directly affect families’ possibilities to access the intervention and thus represent barriers to health equity. A concrete example is that despite continuous efforts to increase fathers’ participation in the home visits, they are often limited due to lack of rights on the job market.

“This vulnerability when it comes to work situations, the fathers are forced to take undeclared work or temporary jobs, and it makes it very hard for them to get a leave from work to be home for our visits.” (Key actor 12)

Another negative systemic influence is observed in the dismantling of services in socioeconomically disadvantaged areas, including the closing down of CHC centres. This is understood to counteract the principle of availability of primary healthcare in the community and restricts access for the most disadvantaged families by forcing them to take on long travels to reach CHC.

“We need to turn this trend to close down all services that represent Swedish society in socioeconomically disadvantaged areas. By closing down we are sort of showing we don’t care about this neighbourhood. We can’t close down open daycare, CHC centre, prenatal clinics. To have these resources present in these areas is of highest importance. I think the home visiting programme, CHC, maternal health care, all this should be a part of a much larger societal model for our disadvantaged areas.” (Key actor 5)

A positive development however is also noted in Stockholm region where many of the areas that are implementing the home visiting programme are experiencing less turnover of staff as well as improved capacity to recruit new professionals. This may be a positive side effect of the programme and was understood to be an important counterforce to the dismantling of CHC in the socioeconomically disadvantaged areas.

#### Strengthening the intervention from a health equity perspective

However, potentials to strengthen the intervention’s capacity to contribute towards more equal health and development for children were identified. The principal strategy would be to ensure that there is continuity of support to families over the whole period of early childhood. This could be achieved by extending the home visiting programme over time, linking it to prenatal services before birth and then continuing with visits until the child reaches 2 or 3 years of age. It was believed that a larger number of home visits over a longer period, involving both parental advisor and CHC nurse would be valuable and needed support for parents in addition to the continuous support by the CHC programme.

“I would like to extend the home visiting programme because it is such a good support for the child during the first 15 months. But then the two-year-old child presents more challenges to the parents, so I believe that many parents need more of this support then. And that maybe they have had very good contact and have received a lot of support from us, but at this point the contact with CHC becomes sparser and they have no contact at all with the parental advisor, and then I perceive that you lose a bit of what you have built during the first 15 months.” (Key actor 9)

Collaborations with the prenatal clinics as well as with local preschools were considered strategic to bridge between the services and promote smooth transfers for families within the system. In addition to these, other actors such as the primary healthcare and citizen service were cited as potential collaboration partners that could strengthen the intervention’s capacity.

## Discussion

Central in Rinkeby’s extended home visiting programme is the qualified team of CHC nurse and parental advisors delivering a situation-based intervention with focus on the well-being of the child and strengthening of parents, where ECD content is presented within the frame of the national CHC programme but adjusted to the current needs and interests of each family. Embedded in the universal CHC programme, the home visiting provides extra time and continuity of professionals in the family’s home where content and work methods add up to a complex intervention composed of health promotion and prevention. The intervention is expected to promote capable and healthy parents with responsive parenting practices, contributing to good health and development in the child. It is also aiming for integration through parents’ accessing the welfare system and having a sense of social belonging. While the programme is believed to provide better conditions for health equity it is also understood to have limited influence, albeit with room for improvement.

### Core components and mechanisms

The programme theory that resulted from the analysis contains five core components which are composed of contents, delivery aspects and characteristics of the home visiting professionals, in line with Beatson et al.’s definition [34]. However, the analysis did not reach the point of producing clear indications of all mechanisms of change between specific components and outcomes as proposed by Weiss [43] or investigating the programme theory’s assumptions of specific Mechanisms of Action that would drive changes or development of expected parenting behaviour [44–46]. Still, albeit not clearly expressed in detail in the programme theory, several aspects of the components and assumptions of mechanisms are discernible and will be discussed as follows.

#### Parental involvement

Parents’ participation is identified in the programme theory as the first step towards intended effects, and this concept might be understood to involve the physical presence as well as the emotional engagement of parents in the programme [47]. The key informants attributed the high levels of enrolment of families in Rinkeby to the intervention’s embeddedness in the universal and highly trusted CHC services that acts as a door opener (see Additional file 4). The programme’s first evaluation report also recognised that the universality was an important aspect in preventing stigmatisation of families and promoting high levels of participation [48], similarly to findings of a literature review of British health visiting [49]. Additionally, several other elements of the core components seem to regard what the key actors believe to be factors that promote family motivation and retention in the programme. The expertise of the professionals, which enables the delivery of complex content that is also flexible, are aspects related to the assumption that delivering services that are adjusted to families’ own perceived needs and wishes will improve engagement and retention. This assumption is also underpinning the central strategy of tailoring services within precision home visiting [21–24]. Further, the professionals in the Rinkeby programme apply facilitation strategies to actively engage parents in the home visits, something explored in a previous study on the programme [33], which is a mechanism of involvement also discussed by Supplee et al. [23]. Finally, Supplee et al. present several aspects related to the provider organisation and community, such as a positive work environment, low staff turnover and existence of referral networks, that are understood to influence family retention [23]. Some of these factors were also identified by the key informants and presented in the Rinkeby programme theory and implementation components and conditions (see additional file 4). One specific aspect to highlight here is the importance of flexibility to adjust for fathers’ work hours [23], an aspect which was also observed as a barrier to fathers’ participation in the Rinkeby programme.

#### Strengthening of parents’ self-efficacy

Another assumed mechanism that may be discerned in the programme theory is the belief that the strengthening approach together with delivery of ECD content will increase parents’ knowledge of child development, which in combination with parental well-being should promote a sense of self-efficacy and lead to responsive parenting practices. This reasoning, further explored in previous studies [33, 50], may be traced back to Bandura’s theory of self-efficacy, where the sources of self-efficacy are a person’s own previous experiences of success and failure; observing others perform a task as a model; praise or critique from others; and the person’s physiological and emotional state [51]. Parenting self-efficacy, from this perspective, regards parents’ self-beliefs in their own capacity and skills to execute parenting responsibilities and tasks [52]. This theory is commonly found as underpinning behaviour change interventions, explored in detail for example in the Human Behaviour Change Project [44, 53]. In this initiative, Bandura’s sources of self-efficacy are identified as Behaviour Change Techniques, or active ingredients, and parental self-efficacy, or “Beliefs about capabilities” is recognized as a Mechanism of Action promoting the desired change in parenting behaviour [44].

#### Building of trust

A final assumption about core components and mechanisms that may be discerned from the programme theory is that information, guidance, encouragement and a trustful relation between professionals and parents should improve parents’ trust in healthcare, social services and the welfare system, and increase access to and use of these resources. The concept of self-efficacy may be observed within this mechanism as well, but in this instance as part of the Health Belief Model, where the belief in the own capacity to overcome barriers is understood to be one of the variables that influence health behaviour [54]. Practical examples from the Rinkeby programme related to barriers to services, such as insecurity and social isolation, have been investigated in a previous study [55]. In addition, the central importance of the establishment of a trustful relation on which the intervention rests, has been widely recognized in the home visiting field [23, 47, 49, 56–59], and research into the concept of trust within the healthcare setting have also indicated how trust on micro and macro levels are interconnected so that a patient’s trust in a provider may positively influence trust in the healthcare institution and thus facilitate the use of such a system [60, 61].

Considering the above discussion of assumptions and possible mechanisms, an important next step would be to further explore and develop the key components and programme theory of the present study, in collaboration with home visiting professionals and key actors.

### Advantages and challenges of a flexible model

The responsive and flexible approach that guides the content and work methods in the core components in the Rinkeby programme implies both advantages and challenges that are relevant to consider. The inherent flexibility allows for the qualified and experienced professionals’ continuous screening and adjustment of content to family needs which may be understood as a strength of the model [49]. However, such an approach also depends on the competencies of the professionals and entails a practice judgement of which the professional is the solely responsible [59]. The broadness of core components and openness to individual tailoring may further be seen as a challenge to ensuring fidelity in the scaling up of the model. However, on the other hand, it gives some room for adjustments to new programme sites and target groups. Still, allowing for continuous individual tailoring may be understood to pose challenges to identify and test the effects of individual Behaviour Change Techniques [44–46] or specific active ingredients as proposed in precision home visiting [21–24].

### Components of evidence-based home visiting

A further relevant exercise in assessing the core components of the Rinkeby home visiting programme is to compare them to reviews of evidence-based home visiting interventions. This effort is not all that straight forward due to the large diversity of home visiting programmes in terms of aims, structure and contexts. However, a recent systematic review of home visiting programmes, presented in two articles by Molloy et al. [11] and Beatson et al. [34], focused on programmes lasting at least 12 months, with content predominantly delivered by nurses or midwives, characteristics that correspond well to the structure of the Rinkeby extended home visiting programme. A number of common process and provider components were found for the seven successful programmes reviewed: antenatal initiation, duration until child is aged 2 years, with approximately 25 visits, carried out by highly qualified or experienced nurses with programme-specific training, receiving regular supervision, recommended caseload of 25 families, continuity of care, and families have access to multidisciplinary support (incl. social workers) [34]. It appears that a number of these components also can be identified in the programme theory of the Rinkeby home visiting programme. The highly qualified and experienced professionals providing continuity of care are central also to the Rinkeby model, and while Beatson et al. introduce social workers through the component of multidisciplinary support [34], in Rinkeby they hold a permanent role on the team with equal participation as the CHC nurse [33]. Regular supervision is another common feature, but in terms of programme-specific training, there is a written guide but no formalised training programme for professionals in the Rinkeby model. Also, in the Rinkeby model, the families have access to multidisciplinary support, through collaboration with local actors as well as through referrals to additional resources facilitated by the home visiting staff.

The Rinkeby intervention’s embeddedness in the CHC and preventive social service structures influence the analysis of caseload as well as dose. The professionals in Rinkeby are not exclusively involved in home visits but also engage in delivery of other services, thus a comparison of caseloads become less meaningful. Further, while the Rinkeby home visiting programme as such consists of six home visits over 15 months, which is considerably less than what is recommended by Beatson et al., it is complemented by the universal CHC programme where the family will participate in approximately 15 additional visits with the same nurse at the CHC centre until the child is six years [8]. Antenatal initiation was recommended by the key actors as a potential improvement of Rinkeby’s intervention, along with a prolonged duration until 2–3 years. This would correspond well to Beatson et al.’s core components.

In terms of content, Beatson et al. identified that all reviewed successful programmes contained maternal and child health, child development and parenting skills, child safety, use of social and community resources, maternal self-sufficiency, as well as a focus on establishing a positive alliance between parents and professionals [34]. All of these are recognized as included in the Rinkeby model, also further detailed in previous studies [33, 50]. The only noted difference is that the Rinkeby model additionally contains an expressed focus on both parents, and while the family’s health, living situation and future plans are present in the content, there is no specific content targeting maternal self-sufficiency [50].

### Towards health equity

The overarching goals of the programme theory are to promote good and equal conditions for healthy development and decrease the social gradient in health. The focus on health equity has been present in the Rinkeby model from the start with the intention to apply the principles of Proportionate Universalism in practice [30]. Proportionate Universalism, as proposed by Marmot et al. contains two central principles: to ensure access to good quality universal services for the whole population; and to guarantee increased support to those with higher needs following the social gradient [5, 32, 62]. Since their introduction, the principles have received criticism for being too vague with regards as to how they should work in practice [17, 49, 63, 64]. Considering the Rinkeby intervention however, it seems possible to identify the principles of Proportionate Universalism underpinning the programme theory. The intervention provides an extra dose of the universal CHC content while also offering extra content and support targeted at specific individual needs [50]. The professionals have skills and time to identify those at risk of “falling behind” and refer them to further services. This is in line with how Cowley et al. also describe the British practice of health visiting as a suitable service for implementing Proportionate Universalism [49]. Further, the Rinkeby programme theory brings intention and focus on families trusting, accessing and using the universal welfare system, where integration and active participation are long-term aspirations. This aspect of Proportionate Universalism seems to be an important complement to the focus on targeting and one which is also recognised by Cowley et al. [49].

To apply Proportionate Universalism in practice, interventions such as home visiting thus depend on the existence of universal welfare services that are accessible and capable of catering for the further needs of the families. Although Proportionate Universalism encompasses strong recommendations for action on the macro-policy level, research has observed that interventions with an expressed health equity or Proportionate Universalism approach, tend to focus on the individual level [17, 65–67]. If we apply Whitehead et al.’s [68] typology of four categories of health equity interventions: (1) strengthening individuals; (2) strengthening communities; (3) improving living and working conditions; and (4) promoting healthy macro-policies, to the Rinkeby programme theory, it is possible to identify the presence of categories 1–3. The Rinkeby intervention’s focus on strengthening of parents and their parenting practices correspond clearly to the first category of the typology. The second category contains strengthening of horizontal as well as vertical social interaction, the former which may be observed in the intervention’s promotion of local parenting networks through the open daycare, while the latter is present in the support towards integration and active citizen participation. The third category of the typology is present through the efforts to improve families’ access to welfare services. So, while observing a health equity perspective in the Rinkeby programme theory which is broader than the focus on only strengthening individuals, it does not address the fourth category of macro-policies.

Whitehead et al. note that forces on the macro-political level that are increasing health inequities will always limit the positive influence of interventions in the other categories [68], something which is supported by authors arguing for the need of including the structural perspective in interventions aiming for health equity [69–71]. These limitations are also identified in the Rinkeby intervention and context. Previous studies have observed how structural aspects, such as housing policies, are having a direct negative influence on the health of families in Rinkeby [55], while the home visiting professionals recognise that their capacity to deal with these issues is very limited [33]. Further, a previous [55] as well as the present study identified barriers to availability and accessibility to the universal welfare system due to the dismantling of local services in socioeconomically disadvantaged areas. The key actors in the present study also perceived that the home visiting programme has limited capacity to influence health equity when observing the structural determinants’ influence over families’ life situations. Availability and accessibility thus seem to be a key issue where systematic efforts linking macro policies to the local level need to be prioritised on the political agenda. To ensure collaborations between parenting support services and welfare services directed towards housing, education and employment may be concrete actions for strengthening the programme’s capacity to contribute towards health equity.

Further possibilities to strengthen the Rinkeby intervention are identified in the extension of the duration in order to reach parents also prenatally and to keep the contact, and especially make use of the parental advisor’s skills, until the child is aged 2–3 years. Increasing collaboration with prenatal clinic, preschool and other actors was also recommended. These views are well in line with Goldfeld et al.’s findings that disadvantage in early childhood can be complex, follow different pathways and children can experience different types of disadvantages at different points in time [72, 73]. They thus called for inclusion of multidimensional and temporal perspectives when developing interventions to combat health inequities in early childhood [72, 73].

The home visiting programme in Rinkeby has been developed from the ground with key participation of the professionals, and it is also the professionals who play central roles in delivering the intervention, taking decisions on what content to present, carrying the responsibility for early detection of extra needs and how to best provide support. At the same time as research has indicated the importance of managers and practitioners gaining a qualified understanding of health inequity and the need for complex interventions [67, 74], the active involvement of practitioners in designing and evaluating interventions has also been stressed as one aspect of future improvements of home visiting in Sweden [75]. It thus seems important to promote continuous dialogue with managers and professionals around the intervention’s core components and programme theory, in the Rinkeby programme as well as in other home visiting initiatives. We believe that the findings of this study may contribute to informing the discussion on the role of home visiting interventions in promoting good and equal conditions for healthy development for all children.

### Strengths and limitations

A large number of documents from a diversity of sources were available for the study which ensured triangulation of information and the possibility to construct a comprehensive understanding of the programme. The possibility to interview knowledgeable key actors from different roles, organisations, programme sites and that participated in all timepoints of the development and implementation processes was also a strength of the study. It would have been valuable to also access the families’ understanding of core components and programme theory, which was not feasible within the limitations of the study. Cultural adaptation was a topic not directly explored in the interview guide and also absent from the programme theory. Specific questions to the key actors and additional input from families regarding culture might have further qualified the programme theory. Access to quantitative data on the implementation of content during home visits at the different programme sites as well as an extended investigation including home visiting staff at all current programme sites would also surely contribute to the quality and trustworthiness of the findings as well as further development of the components and mechanisms of the programme theory.

## Conclusions

The core components of the Extended home visiting programme in Rinkeby correspond well to those of similar evidence-based home visiting interventions, albeit with increased duration and dose as a possibility for strengthening the intervention. The principles of Proportionate Universalism are present in the programme theory and are believed to be fundamental to the intervention’s capacity to contribute to health equity. The programme’s actions on caring for the health and well-being of children and parents and strengthening responsive parenting practices, in combination with promoting access to the universal welfare services and integration into society, are considered important steppingstones towards the overarching goals. Still, the intervention in itself, requires a favourable macro-political environment in the endeavour to balance the structural determinants’ influence on health inequities. Availability of local resources that respond to families’ needs regarding housing, education and employment are important, and the systematic collaboration between the home visiting programme and such services could strengthen the potential to achieve health equity.

## Supplementary Information

**Additional file 1. **Citation list of ECD and home visiting articles and reviews used for construction of matrix in phase 1.

**Additional file 2. **CFIR domains and constructs, and components from ECD and home visiting literature used in Matrix 1.

**Additional file 3. **List of sources used for the document analysis in phase 1.

**Additional file 4. **Description of target group and implementation components and conditions.

**Additional file 5. **Interview guide key informants.

## Data Availability

The datasets generated and analysed during the current study are not publicly available due to the ethical approval (Dnr: 2013/877–31/1 and Dnr: 2019–01,676) which states that the use of interview data should respect anonymity of participants, may only be accessed by the research group, and be stored locked by passwords. The corresponding author can be contacted for further information.
