# Supplementary material for: Towards health equity: core components of an extended home visiting intervention in disadvantaged areas of Sweden
Source: BMC Public Health. 2022 Jun 1;22:1091. doi: 10.1186/s12889-022-13492-3 (PMC9158140; doi:10.1186/s12889-022-13492-3)
Supplement: Supplementary file 1 — Additional file 1. Citation list of ECD and home visiting articles and reviews used for construction of matrix in phase 1. [file 12889_2022_13492_MOESM1_ESM.docx]

**ADDITIONAL FILE** **1. Citation list of ECD and home visiting articles and reviews used for construction of matrix in phase 1**

1. Gomby DS. Home Visitation in 2005: Outcomes for Children and Parents. Invest in Kids Working Paper No. 7. New York: Committee for Economic Development Invest in Kids Working Group; 2005.
2. Duggan A, Portilla XA, Filene JH, Crowne SS, Hill CJ, Lee H. Implementation of evidence-based early childhood home visiting: Results from the mother and infant home visiting program evaluation. Washington, DC: Office of Planning, Research and Evaluation. Administration for Children & Families, US Department of Health and Human Services; 2018.
3. Moore TG, McDonald M, Sanjeevan S, Price A. Sustained home visiting for vulnerable families and children: A literature review of effective processes and strategies. Prepared for Australian Research Alliance for Children and Youth. Parkville, Victoria: Murdoch Childrens Research Institute and The Royal Children’s Hospital Centre for Community Child Health; 2012.
4. McDonald M, Moore T, Goldfeld S. Sustained nurse home visiting for families and children: A review of effective programs. Prepared for Australian Research Alliance for Children and Youth. Parkville, Victoria: The Royal Children’s Hospital Centre for Community Child Health, Murdoch Childrens Research Institute; 2012.
5. World Health Organization, United Nations Children’s Fund, World Bank Group. Nurturing care for early childhood development: a framework for helping children survive and thrive to transform health and human potential. Geneva: World Health Organization; 2018.
6. Aboud FE, Prado EL. Measuring the implementation of early childhood development programs: Measurement of ECD implementation. Annals of the New York Academy of Sciences. 2018;1419(1):249-63.
7. Yousafzai AK, Aboud FE, Nores M, Kaur R. Reporting guidelines for implementation research on nurturing care interventions designed to promote early childhood development. Annals of the New York Academy of Sciences. 2018;1419(1):26-37.
8. Britto PR, Singh M, Dua T, Kaur R, Yousafzai AK. What implementation evidence matters: scaling-up nurturing interventions that promote early childhood development. Annals of the New York Academy of Sciences. 2018;1419(1):5-16.
9. Tomlinson M, Hunt X, Rotheram‐Borus MJ. Diffusing and scaling evidence‐based interventions: eight lessons for early child development from the implementation of perinatal home visiting in South Africa. Annals of the New York Academy of Sciences. 2018;1419(1):218-29.
