# Supplementary material for: Towards health equity: core components of an extended home visiting intervention in disadvantaged areas of Sweden
Source: BMC Public Health. 2022 Jun 1;22:1091. doi: 10.1186/s12889-022-13492-3 (PMC9158140; doi:10.1186/s12889-022-13492-3)
Supplement: Supplementary file 2 — Additional file 2. CFIR domains and constructs, and components from ECD and home visiting literature used in Matrix 1. [file 12889_2022_13492_MOESM2_ESM.docx]

**ADDITIONAL FILE 2.** **CFIR domains and constructs, and components from ECD and home visiting literature used in Matrix 1**

| **CFIR DOMAINS AND CONSTRUCTS** |
| --- |
| **1. INTERVENTION CHARACTERISTICS** |
| A. Intervention source |
| B. Evidence strength and quality |
| C. Relative advantage |
| D. Adaptability |
| E. Trialability |
| F. Complexity |
| G. Design quality and packaging |
| H. Cost |
|  |
| **2. OUTER SETTING** |
| A. Patient needs and resources |
| B. Cosmopolitanism |
| C. Peer pressure |
| D. External policy and incentives |
|  |
| **3. INNER SETTING** |
| A. Structural characteristics |
| B. Networks and communication |
| C. Culture |
| D. Implementation climate |
| D.1. Tension for change |
| D.2. Compatibility |
| c. Relative priority |
| e. Goals and feedback |
| f. Learning climate |
| E. Readiness for implementation |
| a. Leadership engagement |
| b. Available resources |
| c. Access to knowledge and information |
| **4.CHARACTERISTICS OF INDIVIDUALS** |
| A. Knowledge and beliefs about the intervention |
| B. Self-efficacy |
| C. Individual stage of change |
| D. Individual identification with organization |
| E. Other personal attributes |
|  |
| **5. PROCESS** |
| A. Planning |
| B. Engaging |
| a. Opinion leaders |
| b. Formally appointed internal implementation leaders |
| c. Champions |
| d. External change agents |
| C. Executing |
| D. Reflecting and evaluating |
|  |
| **COMPONENTS FROM ECD AND HOME VISITING LITERATURE** |
|  |
| **TARGET GROUP** |
| Sociodemographic characteristics |
|  |
| **Conditions for participation** |
| Risk/need |
| First-time mothers |
|  |
| **HOME VISITORS** |
| Sociodemographics |
| Residing in programme community |
| Training |
| Experience |
| Time spent in programme |
| Personal/social skills |
| Organisational and administrative skills |
|  |
| **PROGRAMME** |
| Goals |
|  |
| **Structure** |
| Nr. of staff |
| Staff recruitment |
| Caseload |
| Conditions of employment |
| Salary |
| Staff turnover |
| Recruitment of participants (antenatal/postnatal) |
|  |
| **Delivery** |
| Onset |
| Duration |
| Dose |
| Intensity |
| Continuity of home visitor |
| Flexibility of settings/hours |
| Combination with other services |
| Translation |
|  |
| **Content** |
| Nature: |
| Information |
| Assistance (psychosocial support, referral) |
| Screening (development, risk) |
|  |
| Theme (Nurturing care): |
| Good health (physical and mental, child and caregiver) |
| Adequate nutrition |
| Opportunities for early learning |
| Responsive caregiving |
| Security and safety |
| Others |
|  |
| Manual-based or not |
| Tailoring of services to family needs (practical issues, background factors) |
| Use of technique or curriculum (e.g. CBT, motivational interviewing) |
| Theoretical framework/Conceptual model |
|  |
| **Approach** |
| Relationship-based (home visitor – family) |
| Strength-based |
| Co-construction with families |
| Cultural consonance programme-family |
|  |
| **Family engagement** |
| Acceptance rate |
| Participation |
| Retention |
| Active involvement |
| Engagement of fathers |
| Engagement of extended family |
|  |
| **Support** |
| Supervision |
| Initial and ongoing training in programme |
| Support material (manual, curricula, worksheets, peer support) |
| Administrative support |
| Communication support |
| Leadership |
