# Supplementary material for: Towards health equity: core components of an extended home visiting intervention in disadvantaged areas of Sweden
Source: BMC Public Health. 2022 Jun 1;22:1091. doi: 10.1186/s12889-022-13492-3 (PMC9158140; doi:10.1186/s12889-022-13492-3)
Supplement: Supplementary file 3 — Additional file 3. List of sources used for the document analysis in phase 1. [file 12889_2022_13492_MOESM3_ESM.docx]

**ADDITIONAL FILE 3. List of sources used for the document analysis in phase 1**

1. Burström B, Marttila A, Kulane A, Lindberg L, Burstrom K. Practising proportionate universalism - a study protocol of an extended postnatal home visiting programme in a disadvantaged area in Stockholm, Sweden. BMC health services research. 2017;17(1):91.
2. Barboza M, Kulane A, Burström B, Marttila A. A better start for health equity? Qualitative content analysis of implementation of extended postnatal home visiting in a disadvantaged area in Sweden. International journal for equity in health. 2018;17(1):42.
3. Tiitinen Mekhail K, Lindberg L, Burström B, Marttila A. Strengthening resilience through an extended postnatal home visiting program in a multicultural suburb in Sweden: fathers striving for stability. BMC public health. 2019;19(1):102-.
4. Burström B, Mellblom J, Marttila A, Kulane A, Martin H, Lindberg L, et al. Healthcare utilisation and measles, mumps and rubella vaccination rates among children with an extended postnatal home visiting programme in a disadvantaged area in Stockholm, Sweden—A 3‐year follow‐up. Acta Paediatrica. 2020;109(9):1847-53.
5. Barboza M, Marttila A, Burström B, Kulane A. Contributions of Preventive Social Services in Early Childhood Home Visiting in a Disadvantaged Area of Sweden: The Practice of the Parental Advisor. Qualitative health research. 2021:31(8):1380–91.
6. Marttila A, Burström K, Lindberg L, Burström B. Utökat barnhälsovårdsprogram för förstagångsföräldrar - samverkan mellan Rinkeby BVC och föräldrarådgivare inom Rinkeby-Kista socialtjänst. Utvärderingsrapport 2015 [Extended child health care programme for first-time parents - collaboration between Rinkeby CHC centre and parental advisors from Rinkeby-Kista social services. Evaluation report 2015]. Stockholm: Karolinska Institutet; 2015.
7. Marttila A, Lindberg L, Burström K, Kulane A, Burström B. Utökat hembesöksprogram för förstagångsföräldrar - samverkan mellan Rinkeby BVC och föräldrarådgivare inom Rinkeby-Kista socialtjänst. Slutrapport utvärdering 2017. [Extended home visiting programme for first-time parents - collaboration between Rinkeby CHC centre and parental advisors from Rinkeby-Kista social services. Final evaluation report 2017] Stockholm: Karolinska Institutet; 2017
8. Burström B. Rättvis vård för barn - utökat hembesöksprogram vid Rinkeby BVC. [Equitable health care for children-extended home visiting programme at Rinkeby CHC centre.] Barnbladet. 2016; 3.16, vol.41.
9. Mellblom J AH, Fredriksson T, Tordai M. Rinkeby hembesöksprogram - ett utökat hembesöksprogram i samarbete mellan barnhälsovården och socialtjänsten. [Rinkeby home visiting programme - an extended home visiting programme in collaboration between CHC and Social services.] Stockholm: Karolinska Institutet; 2018.
10. Nationell primärvårdskonferens. Utökat hembesöksprogram för förstagångsföräldrar med Johanna Mellblom och Hanna Arvidsson [National primary healthcare conference. Extended home visiting programme for first-time parents with Johanna Mellblom and Hanna Arvidsson.] [podcast on the Internet]. Primärvårdspratarna; 2017 [cited 2021 Nov 18]. Available from: https://soundcloud.com/user-304185756/utokat-hembesoksprogram-for-forstagangsforaldrar-med-johanna-mellblom-och-hanna-arvidsson
11. Nationellt kompetenscentrum anhöriga. Konferens Värna våra yngsta. Vad är föräldrarådgivarens roll under hembesöket? [Swedish Family Care Competence Centre. Conference Care for our youngest. What is the parental advisor’s role during the home visit?] [video file] 2019 [cited 2021 Nov 18]. Available from: https://www.anhoriga.se/nkaplay/varna-vara-yngsta-2019/vad-ar-foraldraradgivarens-roll-under-hembesoket-vad-har-utokade-hembesok-med-bhv-betytt-for-socialtjanstens-forebyggande-arbete-/
12. Kommissionen för jämlik hälsa. Nästa steg på vägen mot en mer jämlik hälsa. Slutbetänkande. [Commission on Equity in Health. Final report] (SOU 2017:47) [Internet] Stockholm: Ministry of Social affairs. [cited 2021 Nov 18]. Available from: http://kommissionjamlikhalsa.se/wp-content/uploads/2017/05/sou_2017_47.pdf
13. Tillitsdelegationen. Med tillit växer handlingsutrymmet. Huvudbetänkande. [Delegation on trust. The action space grows with trust. Main report] (SOU 2018:47) [Internet] Stockholm: Tillitsdelegationen. [cited 2021 Nov 18]. Available from: https://www.regeringen.se/49d37c/contentassets/1705dea13e1845d999ce29016897a1ce/med-tillit-vaxer-handlingsutrymmet--tillitsbaserad-styrning-och-ledning-av-valfardssektorn-sou-201847.pdf
14. Jämlikhetskommissionen. En gemensam angelägenhet. Betänkande. [Commission for equality. A common concern. Report.](SOU 2020:46) Stockholm: Jämlikhetsdelegationen. [cited 2021 Nov 18]. Available from: https://www.regeringen.se/rattsliga-dokument/statens-offentliga-utredningar/2020/08/sou-202046/
15. Nationellt kompetenscentrum anhöriga. Konferens Värna våra yngsta. Inledning: Lena Hallengren, Socialminister. [Swedish Family Care Competence Centre. Conference Care for our youngest. Opening: Lena Hallengren, Minister of social affairs] [video file] 2019 [cited 2021 Nov 18]. Available from: https://www.anhoriga.se/nkaplay/varna-vara-yngsta-2019/inledning-lena-hallengren-socialminister/
16. Socialstyrelsen. Podden om utanförskapet och barnen – tidiga insatser ökar tryggheten. [National board of health and welfare. The pod on exclusion and children – early interventions increase security] [podcast on the Internet] På djupet. Stockholm: Socialstyrelsen; 2018. [cited 2021 Nov 18]. Available from: https://www.socialstyrelsen.se/podd/
17. Socialstyrelsen. Podden om stöd till sårbara spädbarnsföräldrar. [National board of health and welfare. The pod on support to vulnerable parents of infants] [podcast on the Internet] På djupet. Stockholm: Socialstyrelsen; 2018. [cited 2021 Nov 18]. Available from: https://www.socialstyrelsen.se/podd/
18. Region Stockholm. Utökat hembesök från BVC (del 1 av 2) Varför det? [Extended home visits from the child healthcare (part 1 of 2) Why?] [podcast on the Internet] BVC podden. Stockholm: Region Stockholm; 2018. [cited 2021 Nov 18]. Available from: https://bvcpodden.fireside.fm/34
19. Region Stockholm. Utökat hembesök från BVC (del 2 av 2) Så går det till. [Extended home visits from the child healthcare (part 2 of 2) This is how it works] [podcast on the Internet] BVC podden. Stockholm: Region Stockholm; 2018. [cited 2021 Nov 18]. Available from: https://bvcpodden.fireside.fm/35
20. SVT Nyheter. Steg för steg – så går hembesöken till. [Swedish Television News. Step by step – this is how the home visits work] [video file] 2019, Mar 14. [cited 2021 Nov 18]. Available from: https://www.svt.se/nyheter/lokalt/stockholm/sa-gar-rinkeby-hembesoksprogram-till
21. SVT Nyheter. Nyblivna föräldrar i Rinkeby får hembesök. [Swedish Television News. New parents in Rinkeby receive home visits] [video file] 2019, Mar 14. [cited 2021 Nov 18]. Available from: https://www.svt.se/nyheter/lokalt/stockholm/musa-och-dottern-safia-har-deltagit-i-rinkeby-hembesoksprogram
22. SVT Nyheter. Efter hembesöken: fler vaccinerar sina barn. [After the home visits: more people vaccinate their children] [video file] 2019, Mar 14. [cited 2021 Nov 18]. Available from: https://www.svt.se/nyheter/lokalt/stockholm/forskaren-om-rinkeby-hembesoksprogram
23. Hammarfeldt E. Lyckad föräldrasatsning i Rinkeby – Göteborg tar efter. [Successful investment in parents in Rinkeby – Gothenburg copies]. SVT Nyheter. [Internet] 2017 Dec 29. [cited 2021 Nov 18]. Available from: https://www.svt.se/nyheter/lokalt/stockholm/lyckad-foraldrasatsning-i-rinkeby-infors-i-goteborg
24. SVT Nyheter. BVC storsatsar i särskilt utsatta områden. [Swedish Television News. Child healthcare makes large investment in especially disadvantaged areas] [video file] 2019 Jan 6. [cited 2021 Nov 18]. Available from: https://www.svt.se/nyheter/lokalt/vast/bvc-storsatsar-i-sarskilt-utsatta-omraden
25. Stockholms stad. Förnyelsepriset. [Internet]. Stockholm: Stockholms stad. [Updated 2021 May 3; cited 2021 Nov 18] Available from: https://start.stockholm/om-stockholms-stad/priser-och-utmarkelser/priser/fornyelsepriset/
26. Pagels, S. Fler hembesök ger barn och föräldrar en bättre start. [More home visits give a better start to children and parents]. Vårdfokus. [Internet] 2016 Apr 4. [cited 2021 Nov 18]. Available from: https://www.vardfokus.se/barn-och-ungdom/fler-hembesok-ger-barn-och-foraldrar-en-battre-start/
27. Pagels S. På golvet föds insikter. [Insights are born on the floor]. Vårdfokus. [Internet] 2016 May 3. [cited 2021 Nov 18]. Available from: https://www.vardfokus.se/yrkesroller/sjukskoterska/a-golvet/
28. Pagels S. Rinkebyprojektet blir kvar – om det finns pengar. [The Rinkeby project will remain- if there is money]. Vårdfokus. [Internet] 2016 Sep 2. [cited 2021 Nov 18]. Available from: https://www.vardfokus.se/barn-och-ungdom/rinkebyprojektet-kan-bli-permanent/
29. Björkman S. Nu tas nästa steg mot jämlik hälsa. [Now the next step is taken towards health equality]. Vårdfokus. [Internet] 2017 Jun 2. [cited 2021 Nov 18]. Available from: https://www.vardfokus.se/yrkesroller/sjukskoterska/nu-tas-nasta-steg-mot-jamlik-halsa/
30. Weilenmann L. Regeringen vill sprida lyckad Rinkebysatsning på BVC. [The government wants to disseminate successful child healthcare investment from Rinkeby]. Vårdfokus [Internet] 2019 Jun 4. [cited 2021 Nov 18]. Available from: https://www.vardfokus.se/yrkesroller/sjukskoterska/regeringen-vill-sprida-lyckad-rinkebysatsning-pa-bvc/
31. Sandström S. Många små insatser krävs mot allt större hälsoklyftor. [Many small interventions are required against increasing health gaps]. Dagensarena [Internet] 2017 Jun 2. [cited 2021 Nov 18]. Available from: https://www.dagensarena.se/innehall/manga-sma-insatser-kravs-mot-allt-storre-halsoklyftor/
32. Tottmar M. Tryggare och friskare barn med hembesök. [More secure and healthier children with home visits]. Dagens Nyheter [Internet] 2015 Nov 7. [cited 2021 Nov 18]. Available from: https://www.dn.se/sthlm/tryggare-och-friskare-barn-med-hembesok/
33. Gustafsson A. Lyckat projekt i Rinkeby med hembesök byggs ut. [Successful project with home visits in Rinkeby is expanded]. Dagens Nyheter [Internet] 2016 Aug 30. [cited 2021 Nov 18]. Available from: https://www.dn.se/sthlm/lyckat-projekt-i-rinkeby-med-hembesok-byggs-ut/
34. Tottmar M. Det bästa med besöken är närkontakt med familjerna. [The best thing with the visits is the close contact with the families]. Dagens Nyheter [Internet] 2017 Jun 27. [cited 2021 Nov 18]. Available from: https://www.dn.se/sthlm/det-basta-med-besoken-ar-narkontakt-med-familjerna/
35. Tottmar M. Hembesök hos bebisar sprids över stan. [Home visits for babies is spread around town]. Dagens Nyheter [Internet] 2018 May 28. [cited 2021 Nov 18]. Available from: https://www.dn.se/sthlm/hembesok-hos-bebisar-sprids-over-stan/
36. Lefvert M. Fler bebisar i Sundbyberg ska få hembesök. [More babies in Sundbyberg will receive home visits] Mitti Sundbyberg [Internet] 2018 May 28. [cited 2021 Nov 18]. Available from: https://www.mitti.se/nyheter/fler-bebisar-i-sundbyberg-ska-fa-hembesok/lmreB!3842771/
37. Sveriges Radio. Svårt för föräldrar att sätta gränser. [Hard for parents to establish limits] [News report on the Internet] P1-morgon. Stockholm: Sveriges Radio; 2018 [cited 2021 Nov 18] Available from: https://sverigesradio.se/artikel/6872045
