# Supplementary material for: Towards health equity: core components of an extended home visiting intervention in disadvantaged areas of Sweden
Source: BMC Public Health. 2022 Jun 1;22:1091. doi: 10.1186/s12889-022-13492-3 (PMC9158140; doi:10.1186/s12889-022-13492-3)
Supplement: Supplementary file 4 — Additional file 4. Description of target group and implementation components and conditions. [file 12889_2022_13492_MOESM4_ESM.docx]

**ADDITIONAL FILE 4.** **Description of target group and implementation components and conditions**

| **Target group** |
| --- |
| The intervention targets socioeconomically disadvantaged areas with high levels of ill-health. In Stockholm region areas are eligible for receiving the intervention based on the Care Need Index (CNI) which is calculated from seven socioeconomic factors. The interviews indicated that some of the programme sites have a larger mix of socioeconomic status among parents, and varied levels of mental health issues between sites was also mentioned. |
| Originally the intervention targeted all first-time parents registered at the CHC centre in an area. Many areas have now also included families where it is the father's first baby (the mother already have children), and families with previous children who are having their first baby in Sweden. |
| The majority of parents have foreign background (born abroad or with two foreign-born parents) although the size of these groups vary somewhat between the sites. There is a large diversity in terms of ethnicity, time spent in country and levels of education. |
| The families experience different forms of vulnerability including housing, economy, social isolation, language, and migration status. |
| Low trust in district administration and authorities is recognized in the populations in the programme sites. |
| **Implementation components for the daily functioning of the programme** |
| **Programme guide -** A programme guide, developed by the professionals in Rinkeby, offers a frame for the content of each visit as well as methodological guidance in the work method. It is available to all professionals and also used during the introduction of new staff. |
| **Picture materials and pedagogical aids -** To deliver the content and facilitate communication with parents the professionals have access to support in form of picture materials and pedagogical aids such as dolls. |
| **Interpreter service -** To have an interpreter present at the visit is often a condition for the intervention to function. This service is offered to all parents that need and wish. |
| **Collaboration with local actors –** Regular local collaborations with open daycare, library and dentists, among others, strengthen the intervention and ensures access to a network of resources for families. |
| **Joint supervision and skills development -** Both groups of professionals in a programme site participate together in external process supervision on a regular basis. Regional seminars for exchange of experiences and skills-development are also organized every semester and serve as important support in the building and maintenance of collaboration between the two professions, as well as promoting a common understanding and programme fidelity among the different sites.  “That you get help and support and training and opportunity for reflection, for example when you are going to talk about violence with the parents, that is not an easy subject to talk about. And the common supervision is so important, to meet and talk about that which is difficult, because it is not always easy to collaborate either. Even though we have done this for many years now, it is still about two persons who should match up together and with the family.” (Key actor 14) |
| **Common routines -** Common planning and working routines for the booking of home visits are conditions for the functioning of the intervention on a daily basis. This responsibility needs to be clearly assigned to specific roles. |
| **Active and supportive local managers -** Local managers play central roles in the functioning of the intervention and its successful integration into daily operations at the CHC centre and preventive social services. Their participation and support represent a key driver in the implementation. Local managers also play an important role in informing and engaging the support of higher levels of the organisations. |
| **Good communication and shared vision between CHC centre and preventive social services -** To foster frequent, clear and open communication and shared visions between the two organisations is of great importance for the collaboration. This is promoted both by the professionals and the local managers. Proximity between the physical location of the organisations is a positively contributing factor.  “So from the management side we need to keep constantly active and make sure the conditions for collaboration are in place. To have a common vision, ensure good communication, to have the mandate for collaboration, that the leadership has structures for this, lots of things. And resources, you need to have plenty of time.” (Key actor 2) |
| **Implementation conditions for sustainability** |
| **Formalisation within organisational structures -** The intervention has been embedded in the national CHC programme since start and this is considered as an important gateway to recruit families. It has also been fundamental in ensuring sustainability, providing a smooth path from being a project to becoming a natural part of the permanent CHC activities. Existence of permanent financing for the programme within Stockholm Region CHC is also considered an important contribution to sustainability.  “That this is integrated into Swedish CHC, which I believe is the cornerstone of our public health, is one of the most important aspects for this to work to implement and function over time.” (Key actor 1)  On the other hand, the lack of specific permanent financing for the intervention within preventive social services is seen as a concern. To establish formal collaboration agreements between the two organisations is also a suggested way of improving conditions for sustainability. Still, it is commonly felt that the programme is well known and supported among the different levels of organisational leadership, all the way up to governmental levels and this is understood to be fundamental for long-term stability. Managers experience that they are supported by their organisations and many also work actively to share information and results as a strategy to maintain the presence of the programme on the organisational agenda. |
| **Evidence-based programme model** – The involvement of practitioners from Rinkeby together with a research team from academia is perceived to render legitimacy to the programme, which has been important for its spread and sustainability. The programme guide, evaluation reports and scientific articles have contributed to the model being considered evidence-based.  “The politicians believe in the extended home visiting. It is very concrete, there is good research on it, there is a model, Rinkeby and their champions have promoted it well. There are now formal rules on how to apply for it and get funding.” (Key actor 2)  The participation of Rinkeby’s professionals in developing the programme guide and their active role as instructors and mentors in new programme sites is an important source of inspiration for colleagues in other areas.  ”Everybody thought it sounded very exciting and we knew the effects it had produced in Rinkeby. We expected it to be a better way to work. So it was not hard to motivate, everyone working here wanted to start the programme.” (Key actor 9) |
| **Policy and services pulling in the same direction** – For the intervention to achieve its effects, be sustainable and contribute towards the overarching goals of improved health equity it is considered fundamental that the general welfare services and resources also are available to families. Along with availability and accessibility, the alignment and collaboration of services is understood to be an important aspect.  “There should be knowledge and acceptance of each other’s work. And preferably some common visions, over the profession borders, to understand that you walk in the same pace towards the same direction.” (Key actor 2)  Already existing local collaboration between CHC and social services and adjacent physical location have been important drivers for the intervention, and family centres that also include prenatal clinics and open daycare are considered ideal platforms for the programme.  The perception of a common system working in the same direction is also understood to be important for strengthening the families’ trust in society.  “To build these bridges and follow the child’s whole way through society, so to speak, and where the parents should see that these resources are here for you, for you and your child. We work with the same goal, in the same direction, for your children’s development, education and for them to become good citizens.” (Key actor 1) |
