# Supplementary material for: Towards health equity: core components of an extended home visiting intervention in disadvantaged areas of Sweden
Source: BMC Public Health. 2022 Jun 1;22:1091. doi: 10.1186/s12889-022-13492-3 (PMC9158140; doi:10.1186/s12889-022-13492-3)
Supplement: Supplementary file 5 — Additional file 5. Interview guide key informants. [file 12889_2022_13492_MOESM5_ESM.docx]

**ADDITIONAL FILE 5. Interview guide key informants**

**Introduction:** Would you like to present yourself? How long have you worked/been involved in Rinkeby extended home visiting programme? alt. When did you work/were you involved in Rinkeby extended home visiting programme? What is/was your role in the programme?

**The programme’s development phase (to be answered by those key informants who took part)**

1. How did you participate in the development and initiation of the programme?
2. Which were the forces that led to the start of the programme?
3. Which favourable prerequisites existed to successfully develop the initiative?
4. What difficulties and challenges had to be overcome?

**The intervention and its components**

1. What does the extended home visiting programme add to the already existing activities of the Child health care centre and Preventive social services?

[If you look at the preliminary programme theory you can observe a description of the main components of the programme and how it is expected to produce results.]

1. Among the different Activities and Resources, are there any components that are more important than others? Which ones? Why?
2. Are there any components that represent larger challenges to realise?
3. What distinguishes Rinkeby extended home visiting programme from other initiatives of parenting support in early childhood? What is flexible and adjustable of the components and what cannot be changed without losing the programme’s identity?
4. How specific are the Activities and Resources for the families in Rinkeby and how well do they also fit target groups in other geographical locations?

**Results**

1. With regards to the Expected results in the preliminary programme theory, can you recognise them from your practical experience of the programme? Which ones? Which ones can you not recognise? Are there other results from the programme not mentioned in the preliminary programme theory?
2. How would you assess the programme’s results so far? What is positive? What is negative? Which factors do you contribute most to the positive/negative results?
3. If you consider the Overarching goals of health equity and equitable conditions for development, what do you consider is the programme’s capacity and possibility to achieve them? What are contributing factors? What are the challenges?
4. Would additional programme components, strategies or interventions be able to strengthen the programme’s potential to contribute towards health equity? Which ones?

**The programme’s implementation**

[We would like to investigate different drivers and challenges that affects (or have affected) the programme and what conditions that need to be in place in order for the programme to function well. I will therefore ask about different areas such as human resources, internal structure and leadership.]

**Staff**

1. What motivates the staff to work in the home visiting programme? What challenges have you experienced with regards to the staff?
2. Does the staff need any specific support in their work with the programme? Which? (supervision, skills development)?
3. What qualities and competencies does the staff need to do a good job in the programme?

**Internal structure/culture**

1. What conditions need to be in place within the organisation for the programme to function well on a daily basis?
2. What internal logistics are important to have in place (joint agenda, meetings, documentation etc.)?
3. What aspects of the internal culture have contributed positively towards the execution of the programme and what have been challenges?

**Leadership**

1. Does your leadership role differ within the home visiting programme from other parts of your organisation’s activities? (To be answered by those who have a manager role)
2. Which are your principal challenges as a manager of the home visiting programmet?
3. How well is the programme recognised and supported further upwards in the organisation’s hierarchy? How important is it to have recognition and support on higher levels? How does one achieve this?

**Collaboration**

1. How well does the collaboration work between the Child health care and the Preventive social services? What contributes towards a good collaboration? What are the challenges? What conditions need to be in place to guarantee a working collaboration between the two?

**External actors**

1. Which other actors outside the two organisations, if any, plays important roles in the programme? In what way are they important? How do you engage them and maintain their engagement?
2. Which other potentially important actors and groups have not yet been involved in collaboration or networking with the programme? Why not?

**Finalizing**

1. How do you think the Rinkeby extended home visiting programme will develop and spread over the coming five years?
